# An increase in VGF expression through a rapid, transcription-independent, autofeedback mechanism improves cognitive function

**DOI:** 10.1038/s41398-021-01489-2

**Published:** 2021-07-08

**Authors:** Wei-Jye Lin, Yan Zhao, Zhe Li, Shuyu Zheng, Jin-lin Zou, Noël A. Warren, Purva Bali, Jingru Wu, Mengdan Xing, Cheng Jiang, Yamei Tang, Stephen R. Salton, Xiaojing Ye

**Affiliations:** 1 Guangdong Provincial Key Laboratory of Malignant Tumor Epigenetics and Gene Regulation, Guangdong-Hong Kong Joint Laboratory for RNA Medicine, Sun Yat-sen Memorial Hospital, Sun Yat-sen University, Guangzhou, 510120 China; 2 Medical Research Center, Sun Yat-sen Memorial Hospital, Sun Yat-sen University, Guangzhou, 510120 China; 3 Guangdong Province Key Laboratory of Brain Function and Disease, Zhongshan School of Medicine, Sun Yat-sen University, Guangzhou, Guangdong 510080 China; 4 Nash Family Department of Neuroscience, Icahn School of Medicine at Mount Sinai, One Gustave L. Levy Place, New York, NY 10029 USA; 5 Friedman Brain Institute, Icahn School of Medicine at Mount Sinai, One Gustave L. Levy Place, New York, NY 10029 USA; 6 Faculty of Forensic Medicine, Zhongshan School of Medicine, Sun Yat-sen University, Guangzhou, 510080 China; 7 Guangdong Province Translational Forensic Medicine Engineering Technology Research Center, Sun Yat-sen University, Guangzhou, 510080 China; 8 Department of Gastroenterology, the Fifth Affiliated Hospital of Sun Yat-sen University, Zhuhai, 519000 China; 9 Departments of Neuroscience and Psychiatry, Icahn School of Medicine at Mount Sinai, Addiction Institute of Mount Sinai, New York, NY 10029 USA; 10 Department of Neurology, Sun Yat-sen Memorial Hospital, Sun Yat-sen University, Guangzhou, Guangdong 510080 China

**Keywords:** Hippocampus, Molecular neuroscience

## Abstract

The release of neuropeptides from dense core vesicles (DCVs) modulates neuronal activity and plays a critical role in cognitive function and emotion. The granin family is considered a master regulator of DCV biogenesis and the release of DCV cargo molecules. The expression of the VGF protein (nonacronymic), a secreted neuropeptide precursor that also belongs to the extended granin family, has been previously shown to be induced in the brain by hippocampus-dependent learning, and its downregulation is mechanistically linked to neurodegenerative diseases such as Alzheimer’s disease and other mood disorders. Currently, whether changes in translational efficiency of *Vgf* and other granin mRNAs may be associated and regulated with learning associated neural activity remains largely unknown. Here, we show that either contextual fear memory training or the administration of TLQP-62, a peptide derived from the C-terminal region of the VGF precursor, acutely increases the translation of VGF and other granin proteins, such as CgB and Scg2, via an mTOR-dependent signaling pathway in the absence of measurable increases in mRNA expression. Luciferase-based reporter assays confirmed that the 3′-untranslated region (3′UTR) of the *Vgf* mRNA represses VGF translation. Consistently, the truncation of the endogenous *Vgf* mRNA 3′UTR results in substantial increases in VGF protein expression both in cultured primary neurons and in brain tissues from knock in mice expressing a 3′UTR-truncation mutant encoded by the modified *Vgf* gene. Importantly, *Vgf* 3′UTR-truncated mice exhibit enhanced memory performance and reduced anxiety- and depression-like behaviors. Our results therefore reveal a rapid, transcription-independent induction of VGF and other granin proteins after learning that are triggered by the VGF-derived peptide TLQP-62. Our findings suggest that the rapid, positive feedforward increase in the synthesis of granin family proteins might be a general mechanism to replenish DCV cargo molecules that have been released in response to neuronal activation and is crucial for memory function and mood stability.

## Introduction

Neuropeptides play critical roles in the modulation of neural activity and synaptic plasticity, which are required for memory formation and emotional behavior. Neuropeptide precursors are sorted into dense core vesicles (DCVs), subcellular compartments that are present in both axons and dendrites, where they are proteolytically processed into peptides. DCVs have an average diameter of 65 nm, as reported in neurons [1, 2]. The release of DCV components into the extracellular space is achieved by constitutive or activity-regulated secretion. Correct packing and sorting of neuropeptides and neurotrophins have a significant impact on cognitive function. A Val66Met substitution in the *BDNF* coding sequence, for example, has been widely studied in the human population. BDNF Met66 carriers show increased risks of developing cognitive impairment and mood disorders as a result of impaired BDNF pro-protein sorting and secretion [3–5].

The roles of DCV biogenesis in learning, memory, and emotion are still largely unknown. Proteins of the extended granin family, including VGF (nonacronymic), chromogranin A (CgA), chromogranin B (CgB), secretogranin 2 (Scg2), and secretogranin 3 (Scg3), are the major components of DCVs that are known to play critical roles in DCV biogenesis, sorting, and regulated secretion [6]. Cellular expression of granin proteins induces DCV biogenesis and increases both DCV numbers and vesicle sizes in fibroblasts, pheochromocytoma cells, and pancreatic beta-cells [7–9]. For example, overexpression of CgA, CgB, or VGF in fibroblasts that lack a classical regulated secretory pathway results in the de novo production of granule-like structures, while gene silencing studies in DCV-containing cells have revealed reciprocal effects on DCV biogenesis, including decreased DCV number and vesicle sizes [7, 10]. In addition to contributions to DCV biogenesis, granin proteins and their processed peptides are also released into the extracellular space in a regulated manner, where they function in energy and glucose homeostasis and cognition [8].

Expression of the *Vgf*, *ChgA*, *ChgB*, and *Scg2* genes has been reported in neurons, while *Scg3* is expressed in both neurons and astrocytes [11–15]. VGF (nerve-growth factor inducible, nonacronymic) was originally identified for its robust induction by nerve growth factor (NGF) in PC12 cells [16, 17]. In recent studies, reduced cerebrospinal fluid (CSF) levels or brain expression of VGF, Scg2, CgB, and CgA proteins have been reported in patients with Alzheimer’s disease (AD), with an estimated decrease in CSF VGF levels at a rate of 10.9% per year in a longitudinal study of patients with AD [18–20]. VGF has been identified as a key modulator that regulates disease-associated gene and protein networks in the brains of patients with AD and mouse models [21, 22]. Prolonged voluntary exercise, stress-like learned helplessness, and the forced swim test (FST) also regulate hippocampal VGF expression in rodent models [23, 24]. VGF and its C-terminal derived peptide TLQP-62 (named for its four N-terminal amino acids and length) are both required for memory formation and the antidepressant efficacy of exercise and ketamine, as evidenced by the genetic ablation of hippocampal VGF or blocking TLQP-62 peptide function by a direct hippocampal infusion of an anti-TLQP-62 neutralizing antibody [25–28]. Despite the critical roles of VGF in modulating cognitive function in normal and diseased brains, the regulation of VGF and other granin family members in this process remains incompletely understood.

In the present study, we found that contextual fear training induced a rapid increase in the levels of VGF and other granin proteins in the dorsal hippocampus without measurable changes in the mRNAs that encode them. In hippocampal slices and cultured neurons, the posttranscriptional induction of VGF and granin protein expression by the VGF-derived peptide TLQP-62 required mammalian target of rapamycin (mTOR) and G-protein coupled receptor (GPCR) signaling. Using in vitro reporter assays, we revealed a repressive role for the *Vgf* 3′UTR in regulating *Vgf* mRNA translation. Importantly, truncation of the *Vgf* 3′UTR resulted in the upregulation of VGF and other granin proteins, along with improved memory formation and resilience to stress-induced depression-like behaviors. Our findings therefore provide new mechanistic insight into the autofeedback role(s) of VGF in the posttranscriptional regulation of granin protein expression, suggesting the pathological importance of dysregulated VGF synthesis in granin protein-mediated DCV biogenesis in neurodegenerative diseases and mood disorders.

## Materials and methods

### Animals

The *Vgf* 3′UTR-truncated mouse line (*Vgf*^Δ/Δ^) was generated as described previously [25]. A 5′ flanking *lox*P site was inserted into the *Vgf* 5′UTR (*Kpn*I site), and a 3′ flanking *lox*P site with a FRT-flanked neomycin cassette derived from p-*lox*P-2FRT-PGKneo was inserted into the *Vgf* 3′UTR (*Xba*I site) [29]. Mice were group housed on a 12 h–12 h light–dark cycle, and chow diet and water were provided ad libitum. All mouse studies were conducted in accordance with the Guide for Care and Use of Experimental Animals, with experimental procedures approved by Institutional Animal Care and Use Committees at Sun Yat-sen University and Icahn School of Medicine at Mount Sinai.

### Culture and treatment of primary cortical neurons, hippocampal neurons, and the hypothalamic cell line N38

The isolation and preparation of primary rat cortical neurons and primary mouse hippocampal neurons are described in the Supplemental Methods. Cortical neurons (14 days in vitro) were treated with the indicated concentrations of TLQP-62 or scrambled SC-62 peptides (Genscript, Piscataway NJ) for 10 min, with or without rapamycin co-treatment (Abcam, Cambridge, MA), and subsequently harvested in ice-cold protein lysis buffer (see Supplemental Methods) or TRIzol reagent (Invitrogen, Carlsbad, CA).

The N38 hypothalamic cell line was grown and maintained as described previously [30]. Briefly, cells were grown in 4.5 g/liter (25 mM) DMEM containing 10% heat-inactivated fetal bovine serum and 1% penicillin-streptomycin, treated with 10 μM TLQP-62, TLQP-21, AQEE-30, or scrambled SC-62 peptide for 10 min and then harvested in ice-cold protein lysis buffer. For co-treatment with different inhibitors, cells were incubated with rapamycin (20 ng/ml, Abcam, Cambridge, MA), actinomycin D (5 μg/ml, Sigma-Aldrich, St. Louis, MO), puromycin (130 μg/ml, Sigma-Aldrich), cycloheximide (40 μM, Sigma-Aldrich), NF499 (10 μM, Sigma-Aldrich) or BIM46187 (10 μM, Sigma-Aldrich) for 30 min before adding TLQP-62 (10 μM) or the scrambled SC-62 peptide (10 μM).

### Western blot analysis (immunoblotting)

Protein lysates were prepared from mouse brain tissues or hippocampal slices by homogenizing the tissue in ice-cold protein lysis buffer. Details of the immunoblotting procedures and antibody dilution factors are described in the Supplemental Methods. Densitometry results were analyzed by ImageJ software.

### RNA extraction, reverse transcription, and quantitative PCR analysis

RNA was extracted from mouse tissues or cells using TRIzol reagent (Invitrogen) according to the manufacturer’s instructions. RNA samples (0.5 μg) were reverse transcribed using iScript reverse transcription Supermix (Bio-Rad, Hercules, CA). Quantitative PCR analysis was performed using first-strand cDNA templates (5–10 ng) and PerfeCTa SYBR Green FastMix (Quanta Biosciences, Gaithersburg, MD). The delta-delta Ct method was used, and relative gene expression was normalized to the mouse *Gapdh* gene. Primer sequences are available upon request.

### RNAscope in situ hybridization

Mouse brains were perfused and postfixed overnight with 4% paraformaldehyde at 4 °C, followed by vibratome sectioning (10 μm thickness; Leica CM 1950). RNA in situ hybridization was performed using RNAscope (Advance Cell Diagnostics (ACD), Hayward, CA) according to the manufacturer’s instructions. Mouse *Arc*, *Vgf*, and *Scg2* RNAscope^®^ probes were designed by and purchased from ACD. The mouse *Vgf* probe targets the region from bases 277–1806 of the *Vgf* sequence (NM_001039385.1), the mouse *Scg2* probe targets the region from bases 20–1136 of the *Scg2* sequence (NM_009129.2), and the mouse *Arc* probe targets the region from bases 23–1066 of the *Arc* sequence (NM_018790.2). For each gene, 20 pairs of ZZ-target probes were designed for each gene.

### Dual luciferase assay

PsiCHECK-2 reporters fused with full-length (1–500 bp) or partial-length (200–500 bp) mouse *Vgf* 3′UTR sequences or fused with mouse *Gapdh* 3′UTRs were transfected into PC12 cells or N38 cells using Lipofectamine 2000 (Invitrogen) 24 h after cell seeding, according to the manufacturer’s recommendations. Rat PC12 pheochromocytoma cells were grown and maintained in RPMI 1640 containing 15% heat-inactivated fetal bovine serum and 1% penicillin-streptomycin. Forty-eight hours after transfection, the cells were washed with PBS and lysed with Passive Lysis Buffer (Dual Luciferase Reporter Assay System, Promega, Madison, WI) for 30 min with continuous shaking. Cell lysates were analyzed using Luciferase Assay Reagent II, immediately followed by quantitation of firefly luciferase activity and the subsequent analysis of Renilla luciferase activity by the addition of Stop & Glo Reagent.

### Contextual fear conditioning (CFC)

Male mice aged 2–3 months were used. Experiments were performed as described previously [7]. Mice were handled for 3 min daily for 5 days. On day 6 (training day), mice were placed in the chamber (30 × 24 × 21 cm; MED Associates, Fairfax, VT) for 3 min for acclimation, followed by a strong training protocol (0.75 mA, two shocks for 2 s each at a 60 s interval) or a weak training protocol (0.3 mA, one shock for 2 s), followed by an additional 30 s in the conditioning chamber before returning them to their home cages. During the memory retention test (24 h after training), freezing behavior was recorded and analyzed by examiners blinded to the mouse genotypes. Freezing was defined as the absence of visible movements and was scored with a 10 s sampling interval. For locomotor activity, mice were placed in a 44 × 44 cm arena, and the total distance traveled (in cm) was recorded for 10 min and analyzed using a video-tracking system (Ethovision 3.0; Noldus Information Technology, Leesburg, Virginia).

### Novel object recognition (NOR) test

The NOR test was performed under bright light. Mice were handled for 2 min daily for 5 days, followed by habituation to the apparatus without objects (36 ×26 × 22.5 cm open box) for 5 min daily for an additional 4 days. For training procedures, mice were placed in the box with two identical objects (Lego blocks or 100-mL beakers) and allowed to explore the objects for 10 min. Olfactory cues were removed by wiping the chamber with 70% ethanol between trials. Twenty-four hours after training, the memory retention test was performed by placing mice in the apparatus and allowing them to freely explore one novel object and one familiar object (counterbalanced) for 5 min. Training and testing trials were video recorded and analyzed by examiners blinded to the genotype to determine the time the animals spent exploring the novel and familiar objects. Object exploration was determined when the mouse touched its nose to the object or its head was oriented toward the object within 1 cm. The discrimination index (D.I. = [time novel−time familiar]/[time novel + time familiar] × 100) represents the relative time spent exploring the novel versus familiar object.

### Forced swim test (FST)

Four-liter beakers containing three liters of water (25 ± 1 °C) were used, and mice were placed in the beakers for 6 min. Behaviors were video recorded and analyzed by examiners blinded to genotypes. The immobility time in the last 4 min was measured and defined as the absence of movement except moves necessary to keep the head above water.

### Tail-suspension test (TST)

Mice were suspended upside down with adhesive tape 40 cm above the floor. The immobility time was recorded and manually counted over the last 4 min during a 6 min test period. Examiners were blinded to the mouse genotypes.

### Open field test (OFT)

The OFT was performed under red light. The total and center travel distances and time spent by mice in the center of an open field arena (44 × 44 cm) over a 10 min testing duration were measured using Ethovision video tracking software (Ethovision 3.0, Noldus Information Technology).

### Subchronic variable stress (SCVS)

Subchronic variable stress was performed as described previously [31]. Briefly, mice went through three different stressors (foot shock, tail suspension, and restraint stress) over six days. On day 1, 100 random mild electric foot shocks (0.45 mA) over 1 h were performed. On day 2, tail suspension stress was performed which lasted for 1 h. On day 3, restraint stress was applied to mice by a 50 ml conical tube for 1 h within home cage. Mice received repeated stressors for the next 3 days in the same order.

### Statistical analysis

Details of the statistical analysis, including the tests used, sample sizes and p values, are indicated in the figure legends. Analyses were performed using GraphPad Prism 7 software. Comparisons between two groups were performed using two-sided Student’s *t* test. For three or more groups, comparisons were performed using one-way analysis of variance (ANOVA) followed by Tukey’s post hoc test. All data are presented as the mean ± s.e.m.

## Results

### Contextual fear memory training induces an acute increase in dendritic granin mRNA levels and protein translation in the dorsal hippocampus

**Fig. 1:**
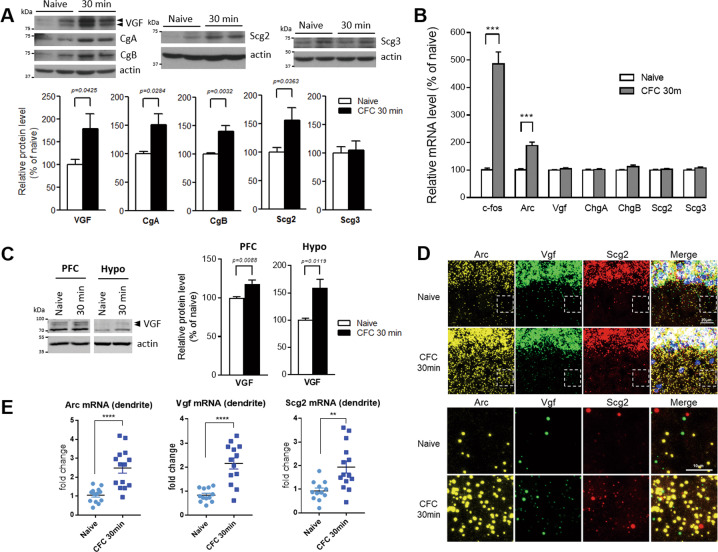
Contextual fear conditioning acutely increases the levels of VGF and other granin proteins and dendritic levels of their mRNAs in the dorsal hippocampus. **A** Increased levels of VGF and other granin proteins in the mouse dHC 30 min after CFC training (0.7 mA, 2 shocks). *N* = 7 (naïve), 8 (CFC 30 min). **B** No changes in *Vgf* or other granin mRNA levels were detected in CFC-trained mice. qPCR showed robust induction of immediate early genes *c-fos* and *Arc* mRNAs in the dorsal hippocampus of CFC-trained mice. *N* = 7 (naïve), 8 (CFC 30 min). **C** Increased levels of VGF protein in the mouse prefrontal cortex (PFC) and hypothalamus (Hypo) 30 min after CFC training (0.7 mA, 2 shocks). *N* = 5 mice per group for PFC, 4 mice per group for hypothalamus. Data in (**A**–**C**) are presented as the mean percent change ± s.e.m. compared to the naïve group and were analyzed using unpaired Student’s *t* test. **D** RNAscope in situ hybridization showed increased signal counts of the *Arc*, *Vgf*, and *Scg2* mRNAs in the dendritic region of the CA1 stratum radiatum in mice after 30 min contextual fear conditioning. Yellow: *Arc* mRNA, green: *Vgf* mRNA, red: *Scg2* mRNA. Scale bars, upper panel: 20 μm, lower panel: 10 μm. **E** Quantification of dendritic mRNA signal counts (large + small) of *Arc*, *Vgf*, and *Scg2* mRNAs. *N* = 4–5 mice per group, 2–4 fields per mouse. Data are presented as the mean fold change ± s.e.m. compared to the naïve group and were analyzed using unpaired Student’s *t* test. **, *p* < 0.01; ***, *p* < 0.001 and ****, *p* < 0.0001.

As shown in our previous study, contextual fear memory formation requires hippocampal VGF expression [25]. We noticed that while VGF protein expression in the dorsal hippocampus (dHC) was already induced at 30 min after contextual fear conditioning (CFC), *Vgf* mRNA levels remained unchanged compared with no shock controls (Fig. 1A, B). Similarly, induction of VGF protein but not its mRNA was also observed in the prefrontal cortex and hypothalamus, two brain areas involved in fear memory formation, in the mice that received training shocks (Fig. 1C and Supplemental Fig. 1). At the same time point, the expression of two immediate early genes associated with memory formation, *c-fos* and *Arc*, was robustly induced (Fig. 1B), indicating that specific induction of VGF protein but not its mRNA may represent a rapid response to neuronal activation during memory acquisition.

VGF belongs to the extended granin family, members of which are known to function in the biogenesis of DCVs. We examined the protein and mRNA levels of other granin proteins at 30 min after contextual fear conditioning (CgA, CgB, Scg2, and Scg3) to determine whether they were regulated in a similar manner to VGF. Interestingly, similar to VGF, levels of the CgA, CgB, and Scg2 proteins, but not the Scg3 protein, were upregulated in the dorsal hippocampus at 30 min after training, while their mRNA levels remained unchanged (Fig. 1A, B). Neural activity is known to regulate the dendritic translocation of *Arc* mRNAs that are involved in synaptic plasticity [32, 33]. We therefore examined the subcellular location of granin mRNAs during memory training in the dorsal hippocampus of naïve and memory-trained mice. In situ hybridization using RNAscope revealed an acute increase in the expression of the *Arc* mRNA in both dendrites and soma of CA1 neurons (stratum radiatum) at 30 min after fear memory training (Fig. 1D, E). Similarly, significant increases in dendritic *Vgf* and *Scg2* mRNA foci were also observed in the neuropil of CA1 neurons 30 min after fear memory training. Therefore, our data revealed that contextual fear training induced the acute translation of granin proteins and increased the localization of dendritic granin mRNAs in the dorsal hippocampus.

### The VGF-derived C-terminal peptide TLQP-62 induces acute VGF and chromogranin B protein translation through a mechanism independent of the BDNF/TrkB signaling pathway

Our previous study showed that the VGF-derived TLQP-62 peptide released from neurons was required for memory consolidation immediately after fear memory training [25]. Notably, direct administration of TLQP-62, but not its scrambled control peptide SC-62, into the cannulated mouse dorsal hippocampus resulted in an acute increase in the expression of the VGF and CgB proteins in the CA1 region 10 min after the TLQP-62 infusion (Supplemental Fig. 2A, B). Consistent with this finding, we observed the acute induction of the expression of VGF and CgB proteins in hippocampal brain slices co-incubated with TLQP-62 (Supplemental Fig. 2C). This autoregulation of VGF protein expression by TLQP-62 was independent of BDNF/TrkB signaling, since blockade of BDNF/TrkB activation by a co-treatment with TLQP-62 and the BDNF scavenger TrkB-Fc failed to repress the TLQP-62-mediated acute induction of VGF and CgB protein expression (Supplemental Fig. 2C). This result indicates that TLQP-62 induces acute increases in the levels of the VGF and CgB proteins through a BDNF/TrkB-independent mechanism [34].

### TLQP-62 mediates acute induction of VGF protein expression in cultured primary neurons and immortalized neuronal cell lines in an mTOR- and GPCR-dependent manner

**Fig. 2:**
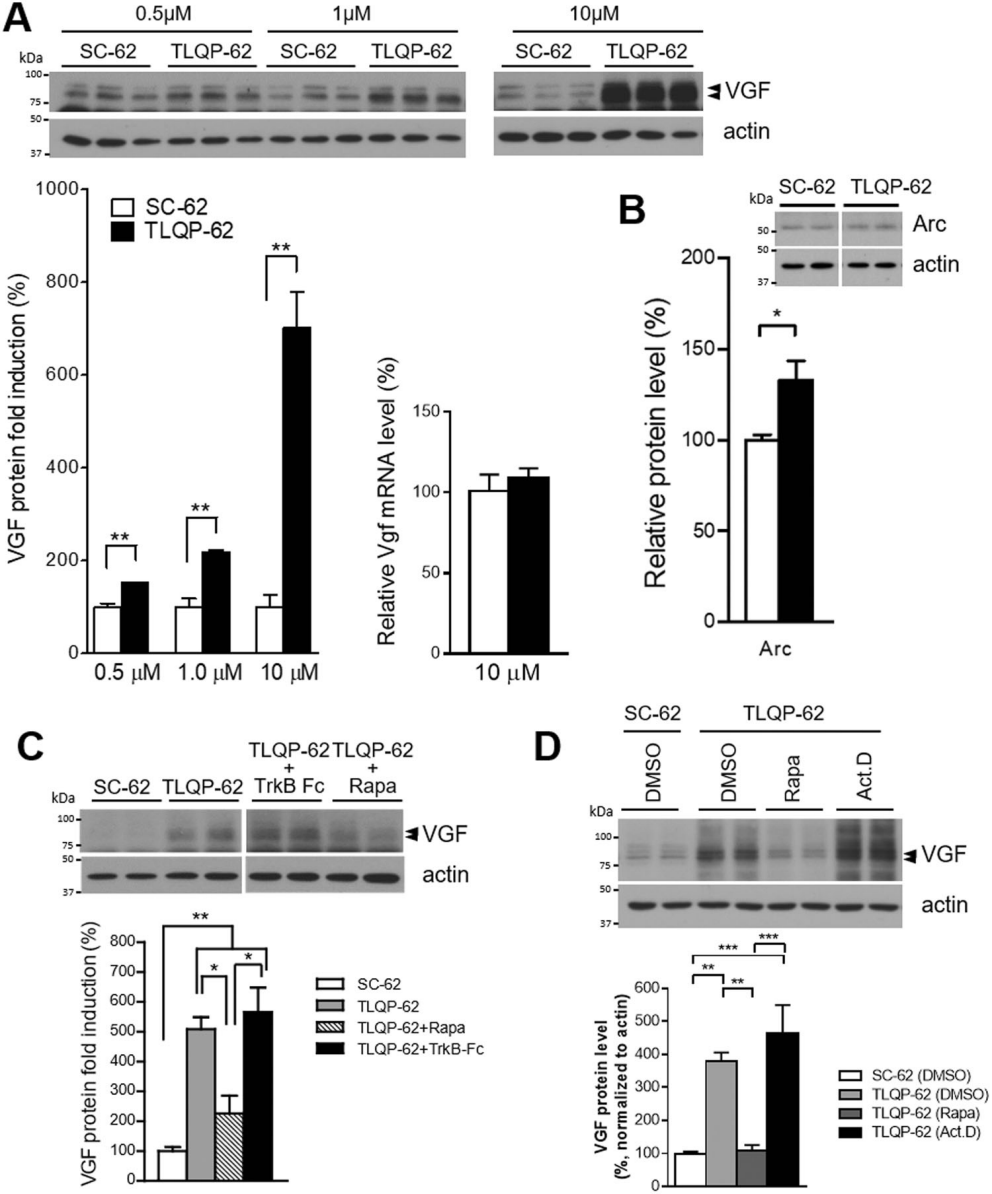
Dose-dependent upregulation of VGF protein translation by the TLQP-62 peptide in cortical neurons depends on mTOR but not TrkB. **A** In primary cortical neurons, the level of the VGF protein was upregulated by TLQP-62 (0.5, 1, and 10 μM) after 10 min of treatment compared with the SC-62 control. No induction of the *Vgf* mRNA was observed in TLQP-62 peptide-treated cortical neurons (10 μM for 10 min). **B** Arc protein levels were upregulated in neurons treated with 10 μM TLQP-62. The densitometry results of immunoblots in (**A**, **B**) are presented as the mean relative percentages ± s.e.m. and analyzed using Student *t* test. *, *p* < 0.05 and **, *p* < 0.01. **C**, **D** The selective increase in VGF protein translation stimulated by TLQP-62 peptide treatment depends on mTOR. **C** The induction of VGF protein expression by TLQP-62 (10 μM for 10 min) was inhibited by the co-treatment of cortical neurons with rapamycin (Rapa, 20 ng/ml) but not TrkB-Fc (0.5 µg/ml). **D** Selective inhibition of mTOR by rapamycin (20 ng/ml) but not the transcriptional inhibitor actinomycin D (Act. D, 5 μg/ml) abolished the TLQP-62-mediated increase in VGF protein levels in the hypothalamic cell line N38. Cells were pretreated with inhibitors, followed by co-treatment with the inhibitors and TLQP-62 (10 μM) for 10 min. Densitometry results for immunoblots are shown as the mean relative percentages ± s.e.m. and were analyzed using one-way ANOVA with Tukey’s post hoc analysis. *, *p* < 0.05; **, *p* < 0.01 and ***, *p* < 0.001.

Activation of mTOR signaling and downstream de novo protein synthesis in the hippocampus are essential for memory consolidation [35]. Primary cortical neurons were treated with the TLQP-62 peptide in combination with the mTOR inhibitor rapamycin to determine whether TLQP-62 regulates the acute induction of VGF protein expression through an mTOR-dependent pathway. We observed a dose-dependent increase in VGF protein levels without a detectable increase in *Vgf* mRNA levels in TLQP-62-treated neurons (Fig. 2A). Notably, the acute increase in Arc protein levels was also observed in neurons treated with 10 µM TLQP-62 peptide (Fig. 2B). Consistent with the results from TLQP-62-treated hippocampal slices, upregulation of VGF protein levels by TLQP-62 in cortical neurons was independent of TrkB signaling, and co-treatment with rapamycin resulted in a complete blockade of the TLQP-62-mediated induction of VGF protein expression (Fig. 2C). Similar to cortical neurons, rapamycin co-treatment resulted in a complete blockade of the TLQP-62-induced increase in VGF protein levels in hypothalamic N38 cells, while blocking transcription by co-treatment with actinomycin D did not exert any effect (Fig. 2D).

**Fig. 3:**
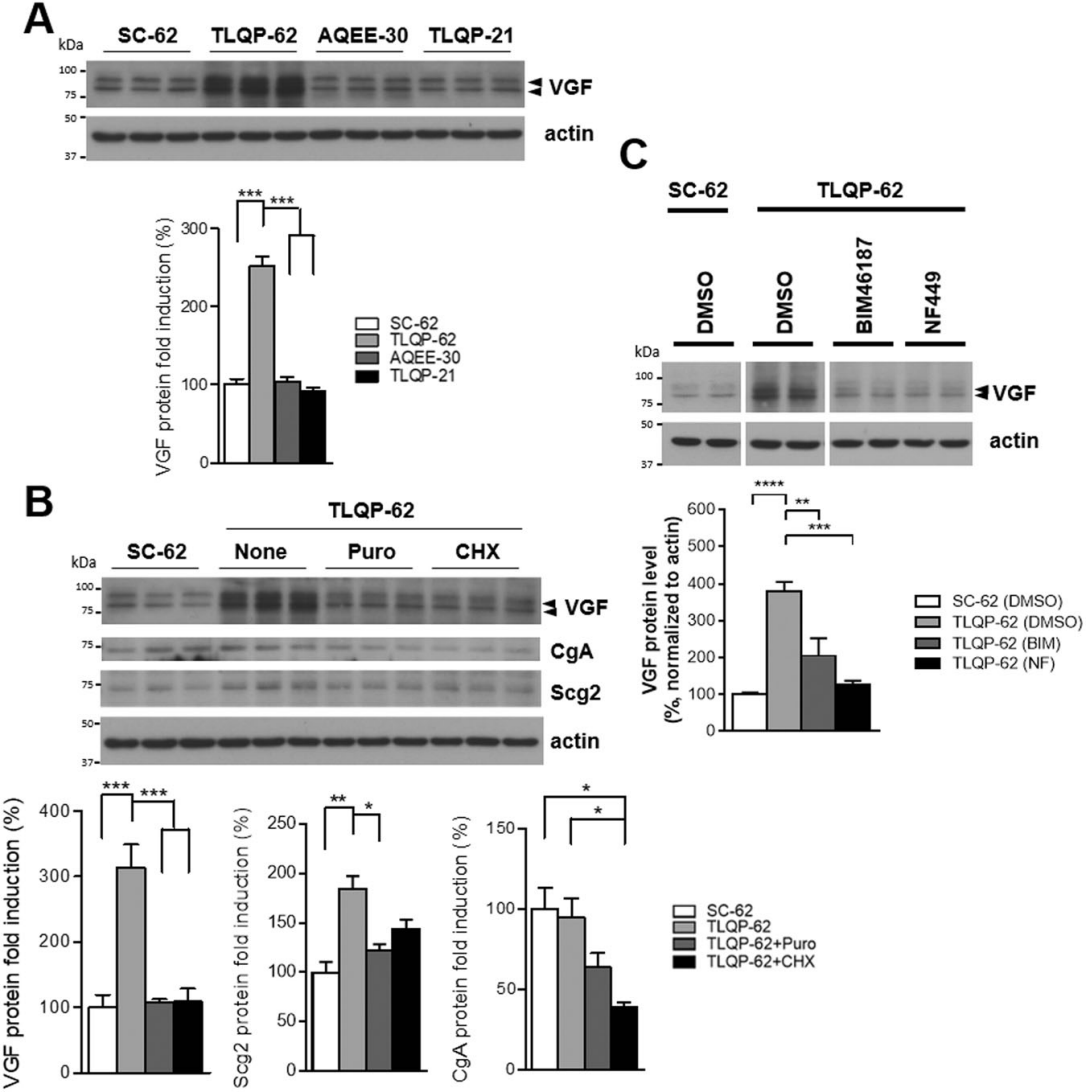
Acute increases in VGF and Scg2 protein levels mediated by VGF-derived TLQP-62 depend on mTOR and GPCR signaling. **A** N38 cells were treated with the indicated peptides (SC-62, TLQP-62, AQEE-30, or TLQP-21, 10 μM) for 10 min. Cells were harvested and analyzed for VGF protein expression using immunoblotting. *N* = 3 per group. Densitometry results are presented as the mean percentage ± s.e.m. and were analyzed using one-way ANOVA with Tukey’s post hoc test. ***, *p* < 0.001. **B** N38 cells treated with TLQP-62 (10 μM) for 10 min showed robust increases in levels of the VGF and Scg2 proteins, while CgA protein levels remained unchanged. Inhibitors of protein translation, puromycin (Puro, 130 μg/ml) and cycloheximide (CHX, 40 μM), each inhibited the TLQP-62-induced increases in VGF and Scg2 protein levels in N38 cells. **C** The selective GPCR inhibitors NF499 (NF, 10 μM, Gαs inhibitor) and BIM46187 (BIM, 10 μM, Gαq inhibitor) abolished the TLQP-62-induced increases in VGF protein levels in the hypothalamic cell line N38. Cells were pretreated with inhibitors, followed by co-treatment with the inhibitor and TLQP-62 (10 μM) for 10 min. Densitometry results are presented as the mean percentage ± s.e.m. and were analyzed using one-way ANOVA with Tukey’s post hoc test. *, *p* < 0.05; **, *p* < 0.01; and ***, *p* < 0.001.

Since TLQP-62 can be processed further into two shorter peptide fragments, TLQP-21 and AQEE-30 [6], we therefore compared whether TLQP-21 or AQEE-30 induced similar effects to TLQP-62 on the induction of VGF protein expression. We applied TLQP-62, TLQP-21, AQEE-30, and the control scrambled peptide SC-62 to N38 cells and determined VGF protein levels 10 min after peptide treatment. We confirmed that only TLQP-62, but not the two shorter peptide fragments derived from TLQP-62, resulted in the acute induction of VGF protein expression in N38 cells (Fig. 3A). Pretreatment with the translational inhibitors puromycin or cycloheximide significantly repressed the TLQP-62-induced increase in VGF protein levels (Fig. 3B). Similar to its actions on VGF, TLQP-62 also increased Scg2 protein levels in N38 cells, a change that was completely abolished by the translational inhibitors puromycin or cycloheximide, while TLQP-62 had no effect on CgA protein levels (Fig. 3B). Our data therefore revealed that the acute increases in VGF and Scg2 protein levels induced by TLQP-62 depended on translation.

G-protein coupled receptors (GPCRs) are the main type of neuropeptide receptors that modulate neuronal activities and synaptic plasticity [36, 37]. Because a specific TLQP-62 receptor has not yet been identified, we tested whether the TLQP-62-mediated regulation of acute VGF translation was GPCR-dependent by cotreating N38 cells with TLQP-62 and NF449 (a Gαs inhibitor) or BIM-46187 (a Gαq inhibitor). Both inhibitors blocked TLQP-62-induced increases in VGF protein levels, indicating that TLQP-62 regulated acute VGF protein expression through a GPCR-dependent mechanism (Fig. 3C). Our findings therefore reveal a novel feedforward, posttranscriptional regulatory mechanism by which a rapid increase in the levels of VGF and other granin proteins is induced by the VGF C-terminal-derived TLQP-62 peptide via an mTOR- and GPCR-dependent signaling pathway.

### Vgf 3′UTR truncation results in the upregulation of neuronal VGF and other granin proteins

**Fig. 4:**
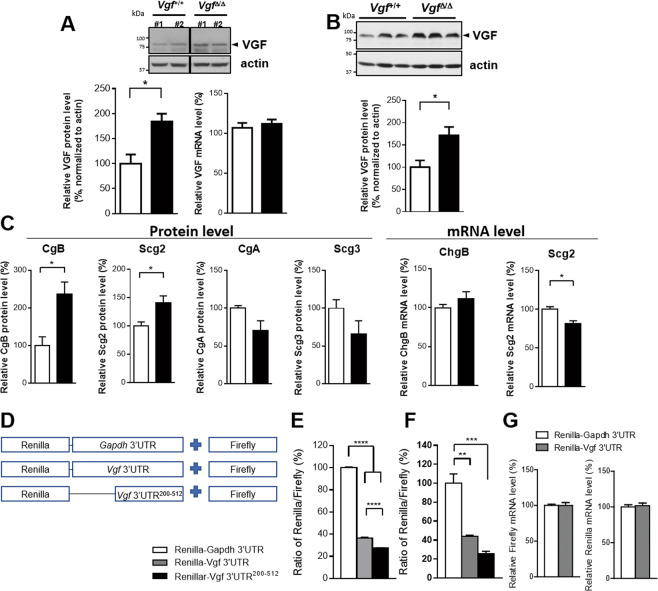
Translation of the *Vgf* mRNA is repressed by its own 3′UTR. **A** Increased VGF protein levels were detected in primary cultured hippocampal neurons derived from homozygous *Vgf* 3′UTR-truncated newborn mice (*Vgf*^Δ/Δ^) compared to wild-type littermate controls (*Vgf*^+/+^). No significant difference in *Vgf* mRNA levels between the two groups was observed. **B** Homozygous *Vgf* 3′UTR truncation (*Vgf*^Δ/Δ^) mice showed increased VGF protein levels in the dorsal hippocampus. *N* = 6/per group for protein measurements. **C** Increased protein but not mRNA levels were observed for other granin family proteins, including CgB and Scg2. Data in (**A–C**) are presented as the mean ± s.e.m. and were analyzed using Student’s *t* test. *, *p* < 0.05 and **, *p* < 0.01. **D**–**G** Repressive effect of the 3′UTR of *Vgf* mRNA on regulating VGF protein translation. **D** Dual luciferase reporter assay. The 3′UTR sequences of mouse *Vgf* or *Gapdh* mRNAs were cloned and fused to the 3′UTR of a Renilla reporter. Firefly luciferase was cotransfected as a control to normalize the transfection efficiency. (**E**) PC12 or (**F**) N38 cells were grown in 12-well plates and transfected with psiCHECK2 plasmids that contained different 3′UTR insertions. Renilla and firefly luciferase activities were measured 48 h after transfection. One-way ANOVA with Tukey’s post hoc test, ***, *p* < 0.001. *N* = 4/group. **G** In N38 cells, comparable mRNA expression levels of the firefly luciferase reporter and Renilla luciferase reporters fused to either mouse *Vgf* 3′UTR or *Gapdh* 3′UTR sequences were measured using qPCR 48 h after transfection.

We previously generated a *Vgf* 3′UTR-truncated mouse model (*Vgf*^*Δ/Δ*^), in which an inverted neomycin selection cassette was inserted into the *Vgf* 3′UTR, creating a noncanonical polyadenylation signal (Supplemental Fig. 3A) and resulting in partial truncation of the *Vgf* mRNA 3′UTR (full-length form: 1–512 bp of *Vgf* 3′UTR; truncated form: 1–202 bp of *Vgf* 3′UTR plus partial sequence of the inverted neomycin selection cassette) (Supplemental Fig. 3B). Interestingly, primary hippocampal neurons (21 DIV) isolated from homozygous *Vgf* 3′UTR-truncated mouse embryos (*Vgf*^*Δ/Δ*^) showed increased VGF protein expression levels (1.84-fold) compared with wild-type neurons, while *Vgf* mRNA levels remained unchanged (Fig. 4A). Moreover, when examining the brain tissues collected from adult *Vgf* 3′UTR-truncated mice (*Vgf*^*Δ/Δ*^), we observed specific increases in the levels of the VGF, CgB and Scg2 proteins but not mRNA levels in the dorsal hippocampus compared with their wild-type littermate controls (Fig. 4B, C). The levels of other granin proteins, including CgA and Scg3, remained unchanged or showed a trend toward a decrease in the dorsal hippocampus of adult *Vgf* 3′UTR-truncated mice. These results confirmed the effect of the *Vgf* 3′UTR on repressing the neuronal expression of VGF and other granin proteins in vivo.

Posttranscriptional regulation of *Bdnf* and *Arc* expression has been reported to be mediated by their 3′UTRs [32, 38]. We subcloned the full-length or 200–512 bp region of the mouse *Vgf* 3′UTR into a Renilla luciferase reporter plasmid (psiCheck2 vector) to investigate whether the *Vgf* 3′UTR may contain *cis*-elements that regulate the translational efficiency of *Vgf* mRNA (Fig. 4D). We found that both the full-length region and the 200–512 bp region of the *Vgf* 3′UTR significantly repressed Renilla protein expression in PC12 pheochromocytoma cells compared with the reporter fused with the mouse *Gapdh* 3′UTR (Fig. 4E). A similar repressive effect of the *Vgf* 3′UTR was also observed in hypothalamic N38 cells (Fig. 4F). Importantly, comparable expression of both *Vgf* 3′UTR-fused Renilla mRNA and *Gapdh* 3′UTR-fused Renilla mRNA was observed in N38 cells, indicating that the *Vgf* 3′UTR repressed translation but not transcription of the Renilla reporter (Fig. 4G). Sequence comparison showed there is a high degree of homology among the human, mouse and rat *Vgf* 3′UTRs, and three potential miRNA target sites including miR-328-3p, miR-27a, miR-423-5p, indicating the evolutionary conservation and functional importance of the *Vgf* 3′UTR, which potentially impacts the regulation of mRNA translation and/or stability (Supplemental Fig. 4). Our findings therefore indicated a repressive role of the *Vgf* 3′UTR in regulating *Vgf* mRNA translation.

### Truncation of the Vgf 3′UTR facilitates long-term memory formation and increases stress resilience

**Fig. 5:**
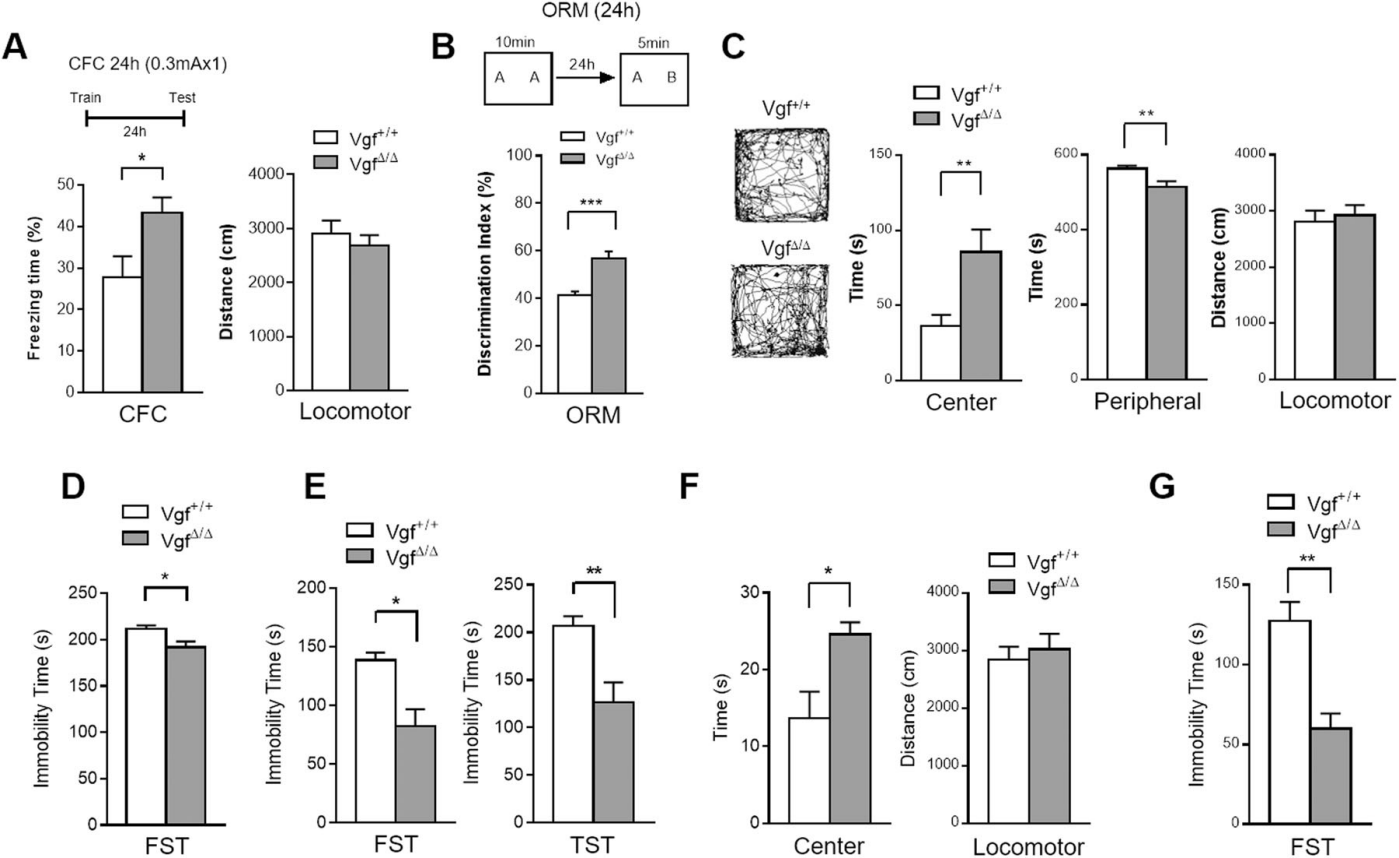
*Vgf* 3′UTR-truncated mice show enhanced memory performance, anxiolytic phenotype, and enhanced stress resilience. **A** *Vgf* 3′UTR-truncated male mice (*Vgf*^*Δ/Δ*^) showed increased freezing behaviors in a hippocampus-dependent CFC test. Mice received mild shock training (0.3 mA), and freezing behavior was tested 24 h later. Locomotor activity remained unaffected. *N* = 4–5 mice per group. Bar graphs present the average freezing (%) ± s.e.m. Data were analyzed using unpaired Student’s *t* test. *, *p* < 0.05. **B** Enhanced novel object memory (discrimination index) in *Vgf* 3′UTR-truncated male mice (*Vgf*^*Δ/Δ*^) compared to wild-type controls (*Vgf*^*+/+*^). N = 8–10 mice per group. The bar graph presents the average discrimination index (%) ± s.e.m. Data were analyzed using unpaired Student’s *t* test. ***, *p* < 0.001. **C** In the open field test, *Vgf* 3′UTR-truncated male mice spent more time in the center of the arena, while locomotor activity (total running distance) remained unchanged compared with wild-type control mice (*Vgf*^+/+^). *N* = 9 mice per group. Data are presented as average time or distance ± s.e.m. and were analyzed using unpaired Student’s *t* test. **, *p* < 0.01. **D** Decreased immobility time of *Vgf* 3′UTR-truncated male mice compared to age-matched control mice in the forced swim test (FST). *N* = 9 mice per group. **E** *Vgf* 3′UTR-truncated male mice preconditioned with a mild foot shock showed an increased difference in immobility time compared with their wild-type littermate controls in the FST and tail suspension test (TST). Mice received mild foot shock (0.3 mA) and were subsequently tested in the FST one week later, followed by the TST 24 h later. *N* = 6–7 mice per group. Data in (**D**, **E**) are presented as average immobility time ± s.e.m. and were analyzed using unpaired Student’s *t* test. **F** *Vgf* 3′UTR-truncated female mice preconditioned with subchronic variable stress (SCVS) showed increased time spent in the center of arena while locomotor activity remained unchanged compared with wild-type control mice (*Vgf*^+/+^). *N* = 4 mice per group. Data are presented as average immobility time or distance ± s.e.m. and were analyzed using unpaired Student’s *t* test. **G** SCVS-preconditioned *Vgf* 3′UTR-truncated female mice showed decreased immobility time compared to age-matched control mice in the FST. *N* = 4 mice per group. Data are presented as average immobility time ± s.e.m. and were analyzed using unpaired Student’s *t* test. *, *p* < 0.05 and **, *p* < 0.01.

Hippocampal VGF expression is required for fear memory formation [25]. Homozygous *Vgf* 3′UTR-truncated mice (*Vgf*^*Δ/Δ*^) and their wild-type littermates were tested to determine whether increased VGF expression in *Vgf* 3′UTR-truncated mice may facilitate memory formation. After exposure to a weak training protocol (0.3 mA, one shock trial), enhanced long-term memory was observed in *Vgf* 3′UTR-truncated male mice (*Vgf*^*Δ/Δ*^) (freezing time (%): *Vgf*^*Δ/Δ*^: 43.3 ± 3.6%; WT: 27.7 ± 5.0%, *p* = 0.038, Student’s *t* test, Fig. 5A), while no difference in long-term memory performance was detected using a strong training protocol (0.75 mA, two shock trial) in different cohorts of male mice (data not shown). Notably, *Vgf*^*Δ/Δ*^ male mice tested for object recognition memory also showed enhanced memory performance (discrimination index (%): *Vgf*^*Δ/Δ*^: 56.6 ± 3.0%; WT: 41.3 ± 1.5%, *p* < 0.001, Student’s *t* test; Fig. 5B), suggesting that the release of 3′UTR-mediated VGF repression, which led to higher VGF protein levels, facilitated long-term memory formation.

VGF expression in the hippocampus is positively correlated with resilient behavior and antidepressant effects, as reported in both mouse and rat models [23, 24, 26, 27]. While a detectable change in the locomotor activity of *Vgf* 3′UTR-truncated male mice was not observed, we noted reduced anxiety, as revealed by increased time spent in the center area of a novel arena (open field test, Fig. 5C). In agreement with previous findings, *Vgf* 3′UTR-truncated male mice (*Vgf*^*Δ/Δ*^) also showed decreased immobility in the FST (Fig. 5D), a stress paradigm commonly used to measure depression-like behaviors and antidepressant efficacy. Interestingly, *Vgf* 3′UTR-truncated male mice that had previously experienced stress (mild foot shock) before the FST and tail-suspension test (TST) showed an increased difference in immobility time compared with their wild-type littermate controls (Fig. 5E), indicating enhanced resilience to stress-induced depressive phenotypes in *Vgf* 3′UTR-truncated mice. In the *Vgf* 3′UTR-truncated female mice, we found no significant difference in open field test and FST performance, although there is a trend of increased time in the center area of a novel arena (Supplemental Fig. 5A, B). Notably, similar to male mice, *Vgf* 3′UTR-truncated female mice that had previously experienced subchronic variable stress (SCVS) showed increased time spent in the center area in the open field test and decreased immobility time in the FST compared with their wild-type littermate controls (Fig. 5F, G). Collectively, these results suggest that a reduction in *Vgf* 3′UTR-mediated inhibition of VGF translation results in enhanced cognition and increased emotional resilience.

## Discussion

In the current study, we identified a posttranscriptional mechanism that acutely regulates the translation of VGF and other granin proteins immediately after contextual fear conditioning or TLQP-62 peptide treatment. Our main findings of the present study are listed below. (1) Contextual fear memory conditioning acutely induced the translation of VGF and other granin proteins in the dorsal hippocampus, prefrontal cortex and hypothalamus without detectable changes in their mRNA levels. (2) The VGF-derived C-terminal peptide TLQP-62 stimulated the acute translation of VGF and other granin proteins via mTOR- and GPCR-dependent mechanisms. (3) The 3′UTR of *Vgf* mRNA regulates its own translational efficiency. (4) Truncation of the *Vgf* 3′UTR resulted in increased VGF protein levels, and (5) mice expressing the *Vgf* 3′UTR truncation showed increased stress resilience and memory performance.

*De novo* protein synthesis in the hippocampus occurs rapidly after learning and is required for memory formation [39]. As shown in a study by Slipczuk et al., intracranial injection of the mTOR-specific inhibitor rapamycin 15 min immediately before memory training dramatically blocks long-term memory [40]. Although neural activity-regulated translation is critical for memory formation, simply increasing new protein synthesis, however, may not be sufficient, as newly synthesized proteins destined for plasma membrane insertion or export from the cell, for example, will also likely require packaging, posttranslational modification, and processing before they are transported to their final cellular destinations where they function or are secreted [10]. For many secretory proteins and their processed peptides, correct and efficient packaging into secretory vesicles (DCVs), together with regulated secretion, are essential for their regulatory roles in synaptic plasticity and memory formation [10, 41]. Of note, expression of exogenous VGF has been shown to induce DCV biogenesis in nonendocrine NIH 3T3 fibroblasts and promote depolarization-induced DCV secretion [7].

Our data further indicated that the expression of DCV component proteins but not their mRNAs was acutely induced in fear memory-associated brain regions, including dorsal hippocampus, prefrontal cortex, and hypothalamus, at 30 min after memory training. Based on accumulating evidence, polyribosomes accompany dynamic changes in the axonal terminal and dendritic spines [42–44], together with rough and smooth endoplasmic reticulum (RER and SER) and *trans*-Golgi-like compartments [45, 46], suggesting that a satellite secretory pathway exists in the axons and spines of neurons. Importantly, for the regulated secretion of neurotrophins and neuropeptides, replenishment and biogenesis of DCVs and efficient packaging of DCV components should determine the biological effects of neuromodulators that are stored in DCVs. Since increased VGF and Scg2 protein production has been reported to facilitate the biogenesis of DCVs and regulate the sorting/secretion of their cargo proteins/peptides [7, 47] and hippocampal VGF expression has been shown to be required for memory formation [25], our findings therefore suggest that the acute production of DCV components such as VGF and Scg2 proteins during memory consolidation may play an essential role in facilitating the sorting and regulated secretion of synaptic plasticity-associated proteins.

We and others have previously shown that hippocampal slices acutely stimulated with VGF-derived TLQP-62 peptide, or intrahippocampal infusion of TLQP-62, both resulted in increased TrkB and CREB phosphorylation, suggesting a downstream BDNF/TrkB/CREB signaling pathway mediated by TLQP-62 [25, 27]. *Vgf* 3′UTR-truncation also resulted in increased TrkB phosphorylation in the dorsal hippocampus and the restoration of long-term memory and neurogenesis in both male and female 5xFAD mice[21]. We therefore reasoned that the memory enhancement, anxiolytic, and antidepressant phenotypes we have observed in the *Vgf* 3′UTR-truncated mice could be explained in part by the increased production/release of TLQP-62 peptide and activation of BDNF/TrkB signaling pathways. Acute antidepressant effects of ketamine have been shown to require hippocampal mTOR activation, suggesting that mTOR-dependent translational events may play a critical role in regulating synaptic plasticity of the neuronal circuits that are involved in antidepressant actions [26]. As shown in our previous studies, hippocampal VGF expression is required for the acute antidepressant efficacy of ketamine and ablation of VGF results in pro-depressant behaviors that are not responsive to ketamine treatment [26, 28]. TLQP-62 administered to the mouse dorsal hippocampus induces acute mTOR activation (phosphorylation) and antidepressant effects, with increased expression of the VGF protein and synaptic α-amino-3-hydroxy-5-methyl-4-isoxazolepropionic acid (AMPA) receptor [26]. Our findings indicate that rapid protein translation and/or secretion stimulated by VGF-derived peptides are integral components of a critical autoregulatory feedback loop that is required for the antidepressant efficacy of ketamine.

The *Arc* and *Bdnf* genes have been extensively studied for their roles in memory-associated synaptic plasticity, and the 3′UTRs of *Arc* and *Bdnf* mRNAs have been found to regulate their subcellular distribution and translation in neurons [32, 38]. The release of 3′UTR-mediated translational repression by mTOR signaling has been reported for the *Arc* mRNA [48], although the underlying mechanism is still unknown. Increased Arc and granin protein expression in the hippocampus of germline VGF-overexpressing (*Vgf*^*Δ/*Δ^) mice and in mouse hippocampal slices treated with the TLQP-62 peptide suggests that VGF and TLQP-62 may fine-tune the mTOR-dependent translation of structural synaptic proteins, which is critical for synaptic scaling and function. Considering the similar behavioral outcomes produced by TLQP-62 administration and the truncation of the *Vgf* 3′UTR, our data further suggest a mechanistic link between neuronal activity-induced TLQP-62 action(s) and the release of translational repression from the *Vgf* 3′UTR.

Translation of mRNA can be regulated by the *cis*-elements that are located in the 3′UTR, which could be targeted either by miRNA-mediated translational repression or directly by RNA-binding proteins (for example, the AU-rich element-binding proteins) [49]. We have completed sequence alignments of *Vgf* 3′UTRs and found high evolutionary conservation between human and rodent *Vgf* 3′UTR sequences, a potential indication of the functional importance of the *Vgf* 3′UTR (Supplemental Fig. 4). Three potential miRNA target sites were found in the mouse *Vgf* 3′UTR (miR-328-3p, miR-27a, miR-423-5p), whose predicted seeding sequences in the 3′UTR showed 100% conservation in human, rat, and mouse. While there is currently no direct evidence supporting the roles of these miRNAs in regulating VGF expression, we note that VGF has been shown previously to enhance oligodendrogenesis by stimulating the proliferation of oligodendrocyte precursor cells (OPCs). Elevated production of intrinsic miR-27a and miR-328-3p has been found in the multiple sclerosis (MS) lesion and mouse MS model, while overexpression of miR-27a led to suppression of OPC proliferation and remyelination [50–52]. These reports suggest a possible mechanistic role of miR-27a and miR-328-3p in regulating VGF expression and remyelination in demyelination-related diseases. To identify the candidate miRNAs that may target the *Vgf* 3′UTR and regulate VGF protein expression, brain regions that show acute VGF protein induction after fear memory training could be examined by RNAseq or directly by qPCR to examine whether the expression levels of three miRNAs negatively correlate with VGF protein levels. Direct experiments that overexpress candidate miRNAs or knock down endogenous miRNAs in neurons could also be undertaken to examine the regulatory roles of candidate miRNAs in the translation of VGF mRNA. Additionally, luciferase reporters fused to the *Vgf* 3′UTR can be used to examine the physical binding of miRNAs to the *Vgf* 3′UTR. Alternatively, selective mutagenesis of seeding sequences could reveal the regulatory effects of miRNAs on the translational efficiency of luciferase reporters.

The essential role that dorsal hippocampal VGF plays in the consolidation of fear memory has been reported previously, in which both the protein and mRNA levels of VGF were induced at 1 h and 6 h after fear memory training [25]. Loss-of-function studies of VGF in the dorsal hippocampus by either genetically ablating VGF expression or by acute administration of anti-TLQP-62 neutralizing antibodies that sequestered the secreted TLQP-62 immediately after CFC training, further confirmed that hippocampal VGF and its C-terminal derived TLQP-62 peptide were essential for fear memory consolidation [25]. TLQP-62 released from activated neurons may further induce the activation of TrkB/CREB signaling pathways through increased intracellular [Ca2^+^] and BDNF secretion [53, 54]. Based on these and current findings, we propose that there exist distinct regulatory mechanisms that upregulate the expression of VGF and other granin family proteins during memory consolidation. In the acute phase immediately after fear memory training (within 30 min after CFC training), translational activation of *Vgf* and other granin mRNAs without alteration in mRNA levels occurs through an as yet unknown receptor and mTOR-dependent mechanism (Supplemental Fig. 6). This is followed by the slower but sustained phase (1–6 h after CFC training) of transcriptional activation of *Vgf* and other granin mRNAs and protein upregulation through a CREB-dependent mechanism [16, 55].

Similar to VGF, both CgB and Scg2 are predominantly expressed in hippocampal neurons of both mice and humans [56–58], which suggests the possible coregulation of these granin proteins, as indicated by our findings. In the majority of differentiated cells, punctate CgB and Scg2 immunolabeling is detected in the Golgi region, along neurites, in growth cones of developing axons, and in the terminals of hippocampal mossy fibers, similar to the pattern of VGF immunofluorescence staining [56, 59]. Additional findings from the RNAseq data reported by the Schuman group also revealed the localization of mRNAs encoding VGF and other granins and their processing enzymes (PC1/3) in the neuropil of the mouse hippocampal CA1 region, although their study did not exclude the possible contribution from interneurons located in the CA1 neuropil [60, 61]. Our data indicate that contextual memory training induces acute increases in the expression of granin mRNAs, including *Vgf* and *Scg2*, in the CA1 neuropil. This acute increase in granin mRNA expression may participate in memory consolidation by increasing DCV synthesis and secretion locally at the synapse. Together with reduced CSF levels or brain expression of the VGF, Scg2, CgB, and CgA proteins in patients with AD [19, 20], these findings suggest a critical role for VGF in maintaining neuronal activity-modulated synaptic plasticity and cognitive function.

It also remains possible that the effects of VGF we observed on cognitive behavior are indirect, as bioactive peptides derived from other granin proteins including CgB and Scg2 that are elevated in the TLQP-62-treated neurons or in the hippocampal tissues after fear memory conditioning could be responsible for the observed changes in memory. In our previous work, we demonstrated that in vivo administration of TLQP-62 neutralizing antibody immediately after fear conditioning abolished long-term memory formation. This finding suggests that neural activity-induced release of TLQP-62 is required for memory formation [25]. The recent finding that Scg2 and its derived bioactive peptides may play a role in regulating novel environment-induced reorganization of hippocampal connectivity also suggests a critical role for Scg2-derived peptides in the modulation of cognitive function [62]. It would therefore be worth examining the possible roles of these and other granin-derived bioactive peptides by administration of specific neutralizing antibodies. The acute induction of VGF and other granin proteins in the dorsal hippocampus could also result from other unknown GPCR *trans*-activators whose secretion may be induced/enhanced after CFC training or TLQP-62 stimulation, but this possibility requires additional investigation.

In summary, our findings reveal a potential role for VGF and its C-terminal peptide TLQP-62 in the acute induction of the expression of DCV granin proteins as a general mechanism for DCV replenishment in neurons that have undergone stimulus-induced secretion in response to neuronal activation. The mechanism by which mTOR activation mediates the release of *Vgf* 3′UTR-mediated repression of VGF remains to be investigated. Studies aiming to identify the RNA-binding factors that are involved in memory training-induced acute translation of *Vgf* and other granin mRNAs should improve our understanding of the underlying mechanisms that regulate synaptic plasticity in healthy brains and those that are compromised in neurodegenerative diseases.

## Supplementary information

Supplemental Material
