# Supplementary material for: An increase in VGF expression through a rapid, transcription-independent, autofeedback mechanism improves cognitive function
Source: Transl Psychiatry. 2021 Jul 8;11:383. doi: 10.1038/s41398-021-01489-2 (PMC8266826; doi:10.1038/s41398-021-01489-2)
Supplement: Supplementary file 1 — Supplemental Material [file 41398_2021_1489_MOESM1_ESM.pdf]

SUPPLEMENTARY INFORMATION

Title:

An increase in VGF expression through a rapid, transcription-independent, autofeedback mechanism improves cognitive function

List of Authors:

Wei-Jye Lin[1,2,3,4,5]*, Yan Zhao[3,6,7], Zhe Li[3,6,7], Shuyu Zheng[3,6,7], Jin-lin Zou[8], Noël A. Warren[9], Purva Bali[4], Jingru Wu[1,2], Mengdan Xing[10], Cheng Jiang[4,5], Yamei Tang[1, 3, 10], Stephen R. Salton[4,5]*, and Xiaojing Ye[3,6,7]*

* Correspondence to linwj26@mail.sysu.edu.cn, stephen.salton@mssm.edu, and yexiaoj8@mail.sysu.edu.cn

SUPPLEMENTAL FIGURES

Supplemental Fig. 1

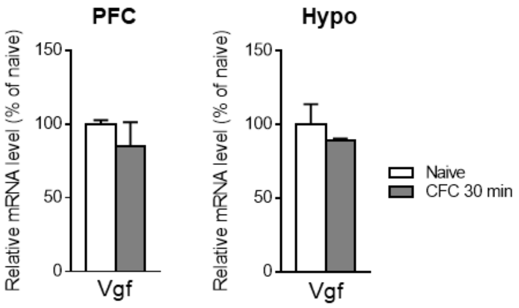

Supplemental Fig. 1. No detectable change of *Vgf* mRNA levels was observed in the prefrontal cortex (PFC) and hypothalamus (Hypo) at 30 min after contextual fear conditioning (0.7 mA, 2 shocks). N=5-6 mice per group (PFC), 4 mice per group (Hypo).

Supplemental Fig. 2

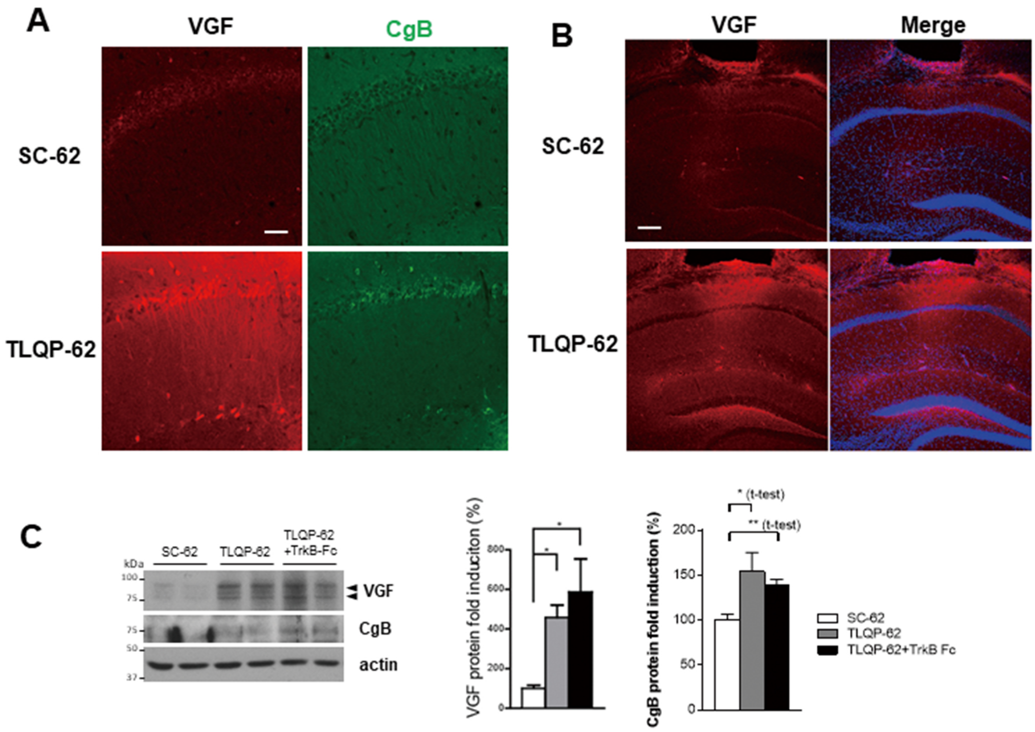

Supplemental Fig. 2. TLQP-62 peptide-mediated acute induction of granin proteins is independent of BDNF/TrkB. (A-B) Mice that received a dorsal hippocampal infusion of the TLQP-62 peptide (0.5 μg peptide in 1 μl), but not the SC-62 peptide, showed robust increases in VGF and CgB protein expression 10 min after the completion of the infusion. (A) Red: antibody detecting the C-terminal region of $VGF_{1\text{-}617}$. Green: antibody detecting the CgB protein. Scale bar: 50 μm. (B) Red: antibody detecting the mid-domain of the $VGF_{1\text{-}617}$ protein. Scale bar: 100 μm. (C) Hippocampal slices were preincubated with TrkB-Fc for 30 min, followed by co-treatment with TLQP-62 or the scrambled SC-62 peptide (10 μM) for 10 min before sample collection. Significant upregulation of both VGF and CgB proteins was observed in TLQP-62-treated slices with or without TrkB-Fc treatment. Data are presented as the mean relative percentage ± s.e.m. and were analyzed using one-way ANOVA with Tukey's post hoc test (lower left panel) or Student's t-test (lower right panel). *, $p<0.05$ and **, $p<0.01$.

Supplemental Fig. 3

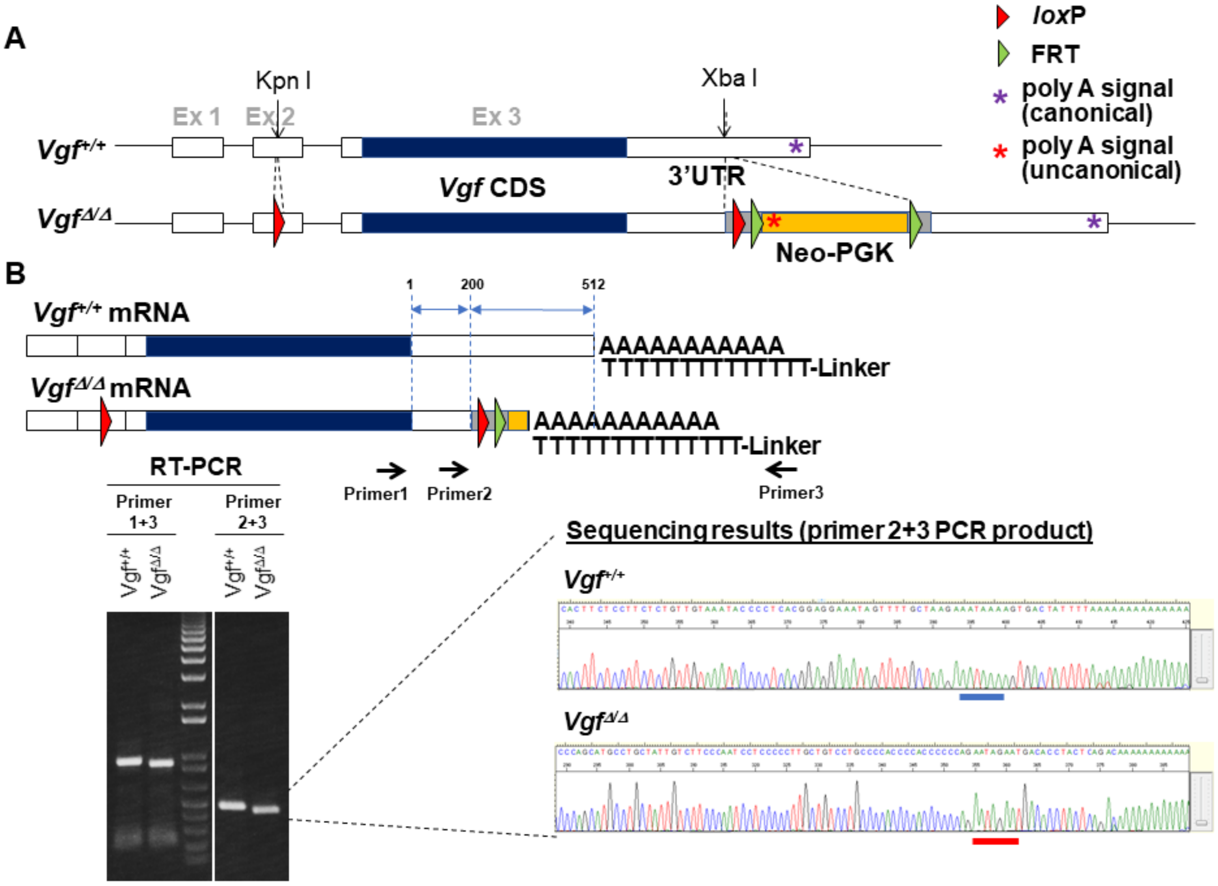

Supplemental Fig. 3. The targeted *Vgf* allele expresses a truncated mRNA product. (A) A PGK-neo cassette containing an uncanonical poly-A signal was inserted into the *Vgf* 3'UTR, and this construct was used to target the *Vgf* gene by homologous recombination. (B) A 3'UTR-truncated mRNA transcript was identified from the floxed VGF allele. Total RNA extracted from wild-type or *Vgf*-floxed homozygous (*Vgf*$^{\Delta/\Delta}$) hippocampal neurons was reverse transcribed with a poly-dT linker. Locations of the PCR primers used in this study are illustrated. A shorter mRNA product transcribed from the *Vgf*-floxed allele, including the 1-201 bp region of the *Vgf* 3'UTR (missing 202-512 bp region) and partial sequence transcribed from the inserted PGK-Neo cassette, was detected using RT-PCR. Lower right panel, sequencing results identified an uncanonical poly-A signal in the PGK-Neo cassette, which resulted in the production of the shorter mRNA transcript from the *Vgf*-floxed allele. The poly-A signal is underlined (blue line: canonical; red line: noncanonical).

Supplemental Fig. 4

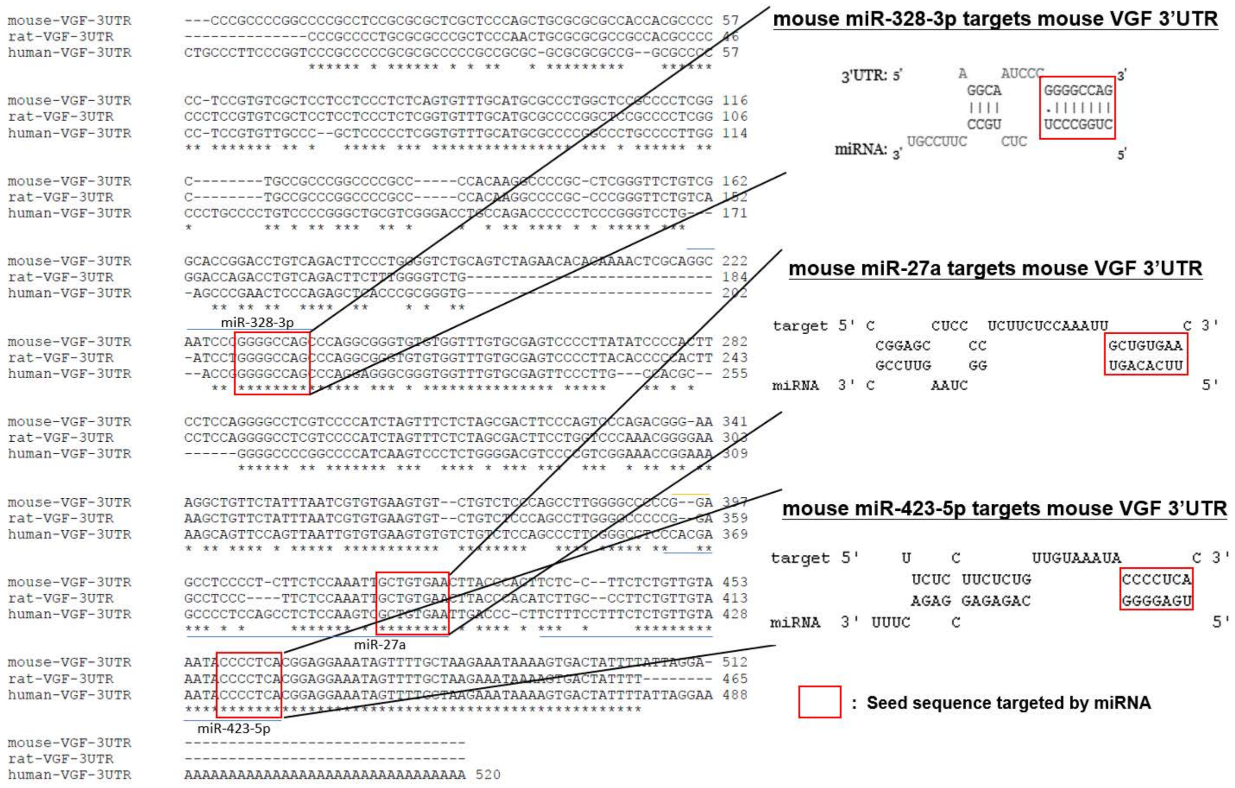

Supplemental Fig. 4. Sequences of mouse, human, and rat *Vgf* 3'UTRs were aligned by ClustalW2 (http://www.ebi.ac.uk/tools/clustalw2). Red box indicates the evolutionarily conserved seed sequence targeted by miR-328-3p, miR-27a and miR-423-5p in the *Vgf* 3'UTR. Blue line indicates the predicted miRNA target region in the *Vgf* 3'UTR.

Supplemental Fig. 5

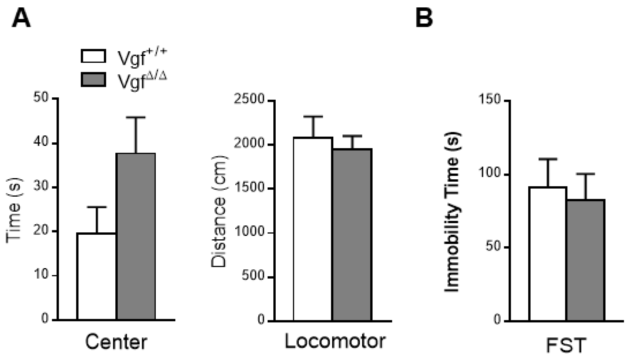

Supplemental Fig. 5. *Vgf* 3'UTR truncation shows a trend in anxiolytic effect in the female mice. (A) In the open field test, *Vgf* 3'UTR-truncated female mice showed a tendency to spend more time in the center of the arena compared with wild-type control mice. Locomotor activity (total running distance) remained unchanged. N=4-5 mice per group. Data are presented as average time or distance ± s.e.m. (B) No difference of immobility time of *Vgf* 3'UTR-truncated female mice compared to wild-type control mice was observed in the forced swim test (FST). N=5 mice per group.

Supplemental Fig. 6

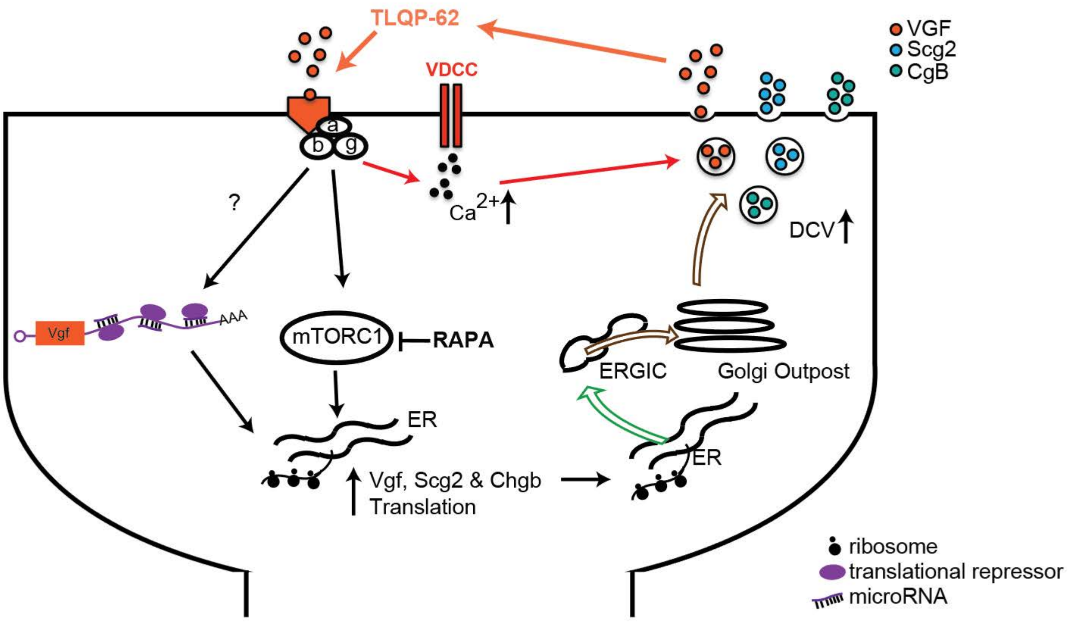

Supplemental Fig. 6. Working model of an autofeedback role of VGF-derived TLQP-62 peptide in inducing rapid translation of VGF and other granin proteins that modulate DCV biogenesis and cognitive function. Neural activity triggers TLQP-62 release from the synaptic terminal, which results in an mTOR-dependent increase of translation of *Vgf*, *Scg2*, and *ChgB* mRNAs via an as yet unknown receptor. Increased granin protein expression facilitates DCV biogenesis. Elevation of intracellular calcium mediated by TLQP-62 further facilitates the exocytosis of DCV and its cargo proteins, resulting in an autofeedback mechanism that modulates synaptic plasticity and cognitive function. VDCC, voltage-dependent calcium channel; RAPA: rapamycin; DCV, dense core vesicle; ER, endoplasmic reticulum; ERGIC, endoplasmic reticulum-Golgi intermediate compartment.

SUPPLEMENTAL METHODS

Stereotaxic cannula implants and peptide injection

Two- to three-month-old male C57BL/6J mice (Charles River Laboratories) were anesthetized with a mixture of ketamine (100 mg/kg) and xylazine (10 mg/kg). For peptide infusion, a double-guide cannula (22-gauge) targeting the bilateral dorsal hippocampus was implanted [AP=-2.0, ML=$\pm$1.5, and DV=-2.0 from bregma (mm)]. After surgery, mice were allowed to recover for 7 d before conducting experiments. For peptide infusion, 1.0 μl (0.5 μg/μl) of the synthetic VGF peptide TLQP-62 or its scrambled peptide control SC-62 (Genscript, Piscataway NJ) was bilaterally infused into the mouse dorsal hippocampus at a rate of 0.2 μl per min, and the needle remained in place for 5 min to prevent backflow.

Immunohistochemistry

Immunohistochemistry was performed as described previously [25]. Briefly, mice were anesthetized with a ketamine/xylazine mixture and intracardially perfused with ice-cold 4% paraformaldehyde (in 1× PBS). Brains were postfixed overnight at 4°C, followed by vibratome sectioning (50 μm thickness; Leica VT 1000S). Free-floating brain sections were permeabilized with 0.2% Triton X-100 in PBS at room temperature and blocked with 3% goat serum and 5% bovine serum albumin (BSA) in PBS containing 0.2% Triton X-100 for 1 h. Brain sections were incubated with an anti-VGF C-terminal (1:1000, rabbit polyclonal Ab[25]), anti-VGF mid-domain (1:500; Santa Cruz Biotechnology, Dallas, TX), or anti-Chromogranin B (1:200; Transduction Laboratories, San Jose, CA) antibodies in 5% BSA overnight at 4°C. On the next day, sections were washed with 0.2% Triton X-100 in PBS and then incubated with goat anti-rabbit Texas Red X (1:500; Life Technologies, Rockville, MD) or goat anti-mouse Alexa Fluor 488 antibodies (1:500; Life Technologies, Rockville, MD) in 1x PBS for 1 h and then washed again. All sections were stained with DAPI, mounted with Prolong Gold antifade solution (Life Technologies, Rockville, MD), and imaged with a Zeiss LSM780 confocal microscope.

Hippocampal slice preparation and treatment

Hippocampal slices (350 μm) were prepared from 2- to 3-month-old C57BL/6J mice as described previously [34]. Slices were perfused with Ringer's solution containing the following (in mM): 125.0 NaCl, 2.5 KCl, 1.3 $MgSO_4$, 1.0 NaH_2PO_4, 26.2 $NaHCO_3$, 2.5 $CaCl_2$, and 11.0 glucose. Ringer's solution was bubbled with 95% O_2/5% CO_2 at 32°C during peptide treatment. Slices were maintained for 1 h before treatment with TLQP-62 (10 μM) or SC-62 (10 μM). For the blocking experiment, slices were preincubated with the TrkB-Fc scavenger (5 μg/ml, R&D Systems, Minneapolis, MN) for 30 min before peptide treatment. Slices were collected at the indicated time point (10 min) and immediately frozen on dry ice.

Culture of primary cortical neurons, hippocampal neurons, and the hypothalamic cell line N38

Primary rat cortical cultures were generated from embryonic day 18.5 embryos. Pregnant Long-Evans dams were euthanized with continuous CO_2 inhalation, and embryos were carefully removed by cesarean section. Cortical tissues were dissected and trypsinized for 15 min at 37°C, washed three times with HBSS and passed through a 40 μm cell strainer to remove debris. Cells were plated in plating media (Minimum Essential Media supplemented with 1 mM sodium pyruvate, 20% glucose, 10% fetal bovine serum, N-2 supplement, and 1% penicillin–streptomycin). On the next day, plating media was replaced with maintenance Neurobasal media (containing 1 mM sodium pyruvate, B-27 and N-2 supplements, and 1% penicillin–streptomycin). On the fourth day *in vitro* (DIV), cells were treated with arabinose-C (5 μM, Sigma-Aldrich, St. Louis, MO), followed by the exchange of half the maintenance media with fresh media every 3 to 4 days. On the 14th day *in vitro*, cells were treated with the indicated concentration of TLQP-62 or scrambled SC-62 peptides and subsequently harvested in ice-cold protein lysis buffer containing 50 mM Tris-HCl (pH 7.5), 140 mM NaCl, 1% Triton X-100, 0.5% Na deoxycholate, 0.1% sodium dodecyl sulfate, and 2 mM EDTA with 1× Halt Protease and Phosphatase Inhibitor Cocktail (Thermo Fisher Scientific, Waltham, MA) or TRIzol reagent (Invitrogen, Carlsbad, CA) for RNA analysis.

Primary culture of mouse hippocampal neurons was performed using homozygous *Vgf* 3'UTR-truncated newborn mice ($Vgf^{\Delta/\Delta}$) or wild-type littermates. Hippocampal neurons were prepared using the method described above for the cortical neuron cultures. Cells were treated with a mitotic inhibitor (Ara-C, 5 μM) on the fourth day *in vitro*, and half of the maintenance media was exchanged

with fresh media every 3 to 4 days. On DIV 21, cells were harvested for protein and RNA extraction.

Western blot analysis (immunoblotting)

Immunoblotting was performed as described previously [25]. Briefly, mouse brain tissues or hippocampal slices were homogenized in ice-cold protein lysis buffer to prepare total homogenates. Protein concentrations were determined using the Bio-Rad protein assay (Bio-Rad Laboratories, Hercules, California), and equal amounts of protein (10 μg per lane for tissue lysates) were resolved on denaturing 10% SDS–PAGE gels and transferred by electroblotting to Hybond-P PVDF membranes (EMDMillipore, Temecula, CA). Membranes were incubated with either an anti-VGF C-terminal (1:1000; rabbit polyclonal[25]), anti-CgA (1:1000; Santa Cruz Biotechnology, Dallas, TX), mouse anti-CgB (1:1000; Transduction Laboratories, San Jose, CA), anti-Scg2 (1:1000; Santa Cruz Biotechnology), anti-Scg3 (1:1000; Santa Cruz Biotechnology), anti-Arc (1:500; Synaptic Systems, Göttingen, Germany), or anti-β-actin (1:5000; EMDMillipore) antibody. The membranes were washed with PBST (0.2% Tween-20 in PBS), incubated with a secondary horseradish peroxidase-labeled donkey anti-rabbit, donkey anti-mouse, or donkey anti-goat antibody (1/6000; GE Health Care Biosciences, Pittsburgh, PA, USA) for 1 h, washed again, and incubated with ECL detection reagents (EMDMillipore). Densitometry analysis was performed using ImageJ software.

SUPPLEMENTAL TABLE 1

qPCR primers: (5'- to -3')

mouse *Vgf*-for:
GGTAGCTGAGGACGCAGTGT

mouse *Vg*f-rev:
GTCCAGTGCCTGCAACAGTA

mouse *Scg2*-for:
AAGAAGAGCAGCTCGAGCAGGCC

mouse *Scg2*-rev:
GGTACTGTCTGTTTGGGGTGTCC

mouse *ChgA*-for:
ACACTTCTGCAGGGCAGC

mouse *ChgA*-rev:
AGTTATTGCAGTTGTGCCCC

mouse *ChgB*-for:
ATTCACCCACAGGCAGAAAG

mouse *ChgB*-rev:
ACAAGTCACGCTAGTCACATGG

mouse *Scg3*-for:
TGTCTCGGCATGCTAGACAC

mouse *Scg3*-rev:
GACGTGGGTTTATTTCCGTG

mouse *c-fos*-for:
CCGAAGGGAACGGAATAAGA

mouse *c-fos*-rev:
TGCAACGCAGACTTCTCATCT

mouse *Arc*-for:
CCAGATCCAGAACCACATGAA

mouse *Arc*-rev:

GAGAGTGTACCCTCACTGTATTG

mouse *Gapdh*-for:
GAACATCATCCCTGCATCCA

mouse *Gapdh*-rev:
CCAGTGAGCTTCCCGTTCA

Firefly luciferase-for:
ATCGTGGACCGCCTGAAGTC

Firefly luciferase-rev:
ACGACGGCGGCAGGCAGC

Renilla luciferase-for:
TGAGGAG TTCGCTGCCTACC

Renilla luciferase-rev:
TGCGGACAATCTGGACGACG
